# Using multivariate endophenotypes to identify psychophysiological mechanisms associated with polygenic scores for substance use, schizophrenia, and education attainment

**DOI:** 10.1017/S0033291721000763

**Published:** 2021-03-18

**Authors:** Jeremy Harper, Mengzhen Liu, Stephen M. Malone, Matt McGue, William G. Iacono, Scott I. Vrieze

**Affiliations:** 1 Department of Psychiatry & Behavioral Sciences, University of Minnesota, Twin Cities, MN, USA; 2 Department of Psychology, University of Minnesota, Twin Cities, MN, USA

**Keywords:** Alcohol use, antisaccade, *δ*, educational attainment, endophenotype, nicotine use, P3, polygenic scores, schizophrenia, *θ*

## Abstract

**Background.:**

To better characterize brain-based mechanisms of polygenic liability for psychopathology and psychological traits, we extended our previous report (Liu et al. Psychophysiological endophenotypes to characterize mechanisms of known schizophrenia genetic loci. Psychological Medicine, 2017), focused solely on schizophrenia, to test the association between multivariate psychophysiological candidate endophenotypes (including novel measures of *θ*/*δ* oscillatory activity) and a range of polygenic scores (PGSs), namely alcohol/cannabis/nicotine use, an updated schizophrenia PGS (containing 52 more genome-wide significant loci than the PGS used in our previous report) and educational attainment.

**Method.:**

A large community-based twin/family sample (*N* = 4893) was genome-wide genotyped and imputed. PGSs were constructed for alcohol use, regular smoking initiation, lifetime cannabis use, schizophrenia, and educational attainment. Eleven endophenotypes were assessed: visual oddball task event-related electroencephalogram (EEG) measures (target-related parietal P3 amplitude, frontal *θ*, and parietal *δ* energy/inter-trial phase clustering), band-limited resting-state EEG power, antisaccade error rate. Principal component analysis exploited covariation among endophenotypes to extract a smaller number of meaningful dimensions/components for statistical analysis.

**Results.:**

Endophenotypes were heritable. PGSs showed expected intercorrelations (e.g. schizophrenia PGS correlated positively with alcohol/nicotine/cannabis PGSs). Schizophrenia PGS was negatively associated with an event-related P3/*δ* component [*β* = −0.032, nonparametric bootstrap 95% confidence interval (CI) −0.059 to −0.003]. A prefrontal control component (event-related *θ*/antisaccade errors) was negatively associated with alcohol (*β* = −0.034, 95% CI −0.063 to −0.006) and regular smoking PGSs (*β* = −0.032, 95% CI −0.061 to −0.005) and positively associated with educational attainment PGS (*β* = 0.031, 95% CI 0.003–0.058).

**Conclusions.:**

Evidence suggests that multivariate endophenotypes of decision-making (P3/*δ*) and cognitive/attentional control (*θ*/antisaccade error) relate to alcohol/nicotine, schizophrenia, and educational attainment PGSs and represent promising targets for future research.

## Introduction

The field of endophenotype research has identified several laboratory-based biobehavioral measures that index the genetic variance related to psychopathology and psychological traits (Anokhin, 2014; Gottesman & Gould, 2003; Gottesman & Shields, 1972; Kendler & Neale, 2010). As detailed in a recent review of psychophysiological endophenotypes (Iacono, Malone, & Vrieze, 2017), candidate endophenotypes for a variety of psychiatric/psychological phenotypes, such as alcohol/substance use and schizophrenia, include measures of spontaneous (resting state) electroencephalogram (EEG) power, P3 event-related potential (ERP) amplitude and *θ* and *δ* oscillatory activity during a target/oddball task, and antisaccade eye-tracking error rate. Prior work has demonstrated that many of these measures show familiarity, that is, they are (a) heritable, (b) are present in close relatives (e.g. offspring or co-twins/siblings) of those with the phenotype or disorder, and (c) share genetic variance with the phenotype/disorder (e.g. overlap between latent genetic factors through biometric modeling of twin/family data) as reviewed in (Anokhin, 2014; Iacono et al. 2017). However, for a measure to be considered a ‘full’ (rather than ‘candidate’) endophenotype, it is expected to show associations with identified genetic variants (Iacono et al. 2017). Despite decades of research following the initial seminal psychiatric endophenotype work of Gottesman and Shields (1972), the ability of candidate endophenotypes to identify robust and reliable specific genetic variants has remained elusive.

Initial conceptualizations of endophenotypes assumed that they were highly heritable but with a simpler genetic architecture than that of psychiatric/psychological phenotypes, which, in theory, would aid substantially in the discovery of associated genes. This does not appear to be the case. We previously evaluated the genetic basis of a collection of promising candidate psychophysiological endophenotypes, including resting-state EEG power; P3 and *θ*/*δ* activity; and antisaccade performance, in a community-based sample of over 4800 individuals and failed to find robust and, convincing evidence for, single genes or variants influencing any of the endophenotypes (Iacono, Malone, Vaidyanathan, & Vrieze, 2014; Malone et al. 2014a, 2014b; Malone, McGue, & Iacono, 2017; Vaidyanathan et al. 2014; Vrieze et al. 2014b). Work using meta-analytic techniques and analysis of analog biobehavioral measures in model organisms (Flint & Munafò, 2007) supports the notion that the effect sizes for genetic variants contributing to endophenotypes are not in fact larger than those contributing to other psychiatric/psychological traits. Like other complex traits, endophenotypes commonly used in behavioral sciences appear to be highly polygenic in nature and at present may not be helpful for discovering genetic variants with large effects.

A promising, yet underappreciated, the utility of endophenotypes is their ability to characterize biological mechanisms related to psychological phenotypes and the genetic variants of these phenotypes as identified through large-scale genome-wide association studies (GWAS). Recent advances in molecular genetics provide evidence that common variants with small effect sizes additively contribute to the phenotypic expression of many psychiatric disorders and psychological traits (Bogdan, Baranger, & Agrawal, 2018). Rather than testing single genes or variants in isolation, polygenic scores (PGS), also known as polygenic risk scores, are a way to model the aggregate influence across genetic variants associated with a phenotype. PGS are calculated by weighting single-nucleotide polymorphisms (SNPs) by the strength of their association with a phenotype (e.g. schizophrenia). Although endophenotypes may not strongly relate to single genes or variants, they may prove useful in understanding the biobehavioral mechanisms of aggregate polygenic liability.

In that vein, our previous paper (Liu et al. 2017) focused on testing the one-to-one association between candidate endophenotypes and a PGS based on a large schizophrenia GWAS meta-analysis by the Schizophrenia Working Group of the Psychiatric Genomics Consortium (2014). Despite relatively well-powered tests, we found no significant correlations after multiple comparison adjustment.

The predictive strength of a PGS is highly dependent on a strong and well-powered discovery GWAS to provide accurate and precise weights for the SNP-phenotype association (Dudbridge, 2013); the lack of significant findings in our previous work may have been in part due to PGS imprecision. Increasingly, well-powered summary statistics identifying more significant reliable effect alleles for a phenotype may improve the statistical power of a PGS to relate to biobehavioral mechanisms. Moreover, while our previous report focused on schizophrenia polygenic risk, it is unknown whether endophenotypes index the polygenic liability for other psychological phenotypes, such as substance use (Liu et al. 2019; Pasman et al. 2018) or educational attainment (Lee et al. 2018). A ‘multivariate endophenotype’ approach, leveraging the covariation across many endophenotypes using statistical techniques like principal component analysis (PCA), may also improve power to detect polygenic effects (Gilmore, Malone, & Iacono, 2010; Harper, Malone, & Iacono, 2019a; Iacono, Carlson, & Malone, 2000; Jones et al. 2006; Price et al. 2006). Multivariate endophenotypes have potentially greater explanatory power than any single endophenotype because they combine the unique and shared (genetic) variance explained across many endophenotypes and help capture the multiple biological and cognitive pathways giving rise to a single phenotype (Gottesman & Gould, 2003).

In the current study, we sought to extend our previous work (Liu et al. 2017) by performing a comprehensive test on the association between multivariate endophenotypes and a range of PGSs, including alcohol/nicotine/cannabis use [the most commonly used substances in the United States; (Substance Abuse and Mental Health Services Administration, 2020)], educational attainment, and an updated PGS for schizophrenia in a large community-based sample of over 4800 individuals from the Minnesota Twin Family Study (MTFS). PCA-based multivariate endophenotypes were derived from 11 psychophysiological measures with reasonable construct validity as candidate endophenotypes for phenotypes of interest (see the subsection ‘Endophenotypes’ of section ‘Methods’), namely, P3 amplitude, band-limited resting-state EEG power, and antisaccade performance [i.e. those studied in Liu et al. (2017)], plus additional measures of *δ* and *θ* oscillatory activity elicited during a visual oddball task (Malone et al. 2017). PGS were derived from the summary statistics of four recent largest of their kind GWAS meta-analyses: (1) alcohol (drinks per week) and nicotine (regular smoking initiation) use (Liu et al. 2019); (2) lifetime cannabis use (Pasman et al. 2018); (3) years of educational attainment (Lee et al. 2018); and (4) an updated GWAS meta-analysis of schizophrenia (Pardiñas et al. 2018). PGSs were calculated using a novel approach, LDPred (Vilhjálmsson et al. 2015), which takes into account the linkage disequilibrium between markers and is a potentially more powerful analytic tool than the traditional PGS calculation approach used in our previous report. To verify the appropriateness of this sample to evaluate PGS-endophenotype relationships, additional tests evaluated measurement construct validity (e.g. heritability of psychophysiological measures, significant associations between PGSs for phenotypes with previously demonstrated genetic correlations). Significant findings would provide important information regarding potential biobehavioral mechanisms related to the polygenic architecture of these psychiatric/psychological phenotypes.

## Methods

### Participants

Participants were assessed as part of the Minnesota Center for Twin and Family Research (MCTFR), a community-based sample of twins and their parents. The reader is referred to our previous papers for extensive details on this sample and the endophenotypes used here (Iacono et al. 2014; Liu et al. 2017; Malone et al. 2017). Participants completed a battery of psychiatric assessments, self-report questionnaires, and behavioral/psychophysiological laboratory-based tests (Iacono et al. 2014, 2017; Iacono & McGue, 2002; Keyes et al. 2009; Wilson et al. 2019). Participants were genotyped on the Illumina 660W-Quad as described previously (Miller et al. 2012; Vrieze et al. 2014a) and then imputed to the Haplotype Reference Consortium (McCarthy et al. 2016) panel using the Michigan imputation server (Das et al. 2016). The number of individuals with genotypes and at least one psychophysiological measure was 4905. We selected individuals primarily of European descent for the current report by calculating four principal components (PCs) on the European population in the 1000 G (1000 Genomes Project Consortium et al. 2015) using PLINK (Chang et al. 2015), projecting the MCTFR genotypes on the resulting PC weights, and selecting participants who fell within the space defined by the 1000 Genomes EUR-ancestry individuals; this resulted in a total sample of 4893 individuals.

### Endophenotypes

The endophenotypes have been described in detail elsewhere (Iacono et al. 2014; Liu et al. 2017; Malone et al. 2017; Vrieze et al. 2014b). All endophenotypes were corrected for sex, age, assessment cohort, and 10 PCs reflecting the major dimensions of genetic variation in this sample, and, for EEG measures, recording system. A brief overview is as follows.

### Event-related EEG

Participants completed a rotated heads visual oddball task (Begleiter, Porjesz, Bihari, & Kissin, 1984) during EEG recording. We focused on EEG activity to target/oddball stimuli. *P3 amplitude* was calculated from the trial-averaged ERP across all target trials at midline parietal electrode Pz. Given the strong evidence that P3 is not a unitary phenomenon but rather a mixture of superimposed *δ* and *θ* frequency-band activity (Karakas, Erzengin, & Basar, 2000a, 2000b; Kolev, Demiralp, Yordanova, Ademoglu, & Isoglu-Alkaç, 1997), we calculated four additional measures [not examined in our prior report (Liu et al. 2017)]. As described in (Malone et al. 2017), we calculated *time–frequency energy (total power)* and *inter-trial phase clustering/coherence* (*ITPC*: a measure of the consistency of the EEG oscillatory signal across trials) for *θ* (at frontal midline electrode Fz) and *δ* (at parietal midline electrode Pz). P3 amplitude reduction is reliably associated with schizophrenia and several forms of substance use, including alcohol, nicotine, and cannabis (Anokhin et al. 2000; Bramon, Rabe-Hesketh, Sham, Murray, & Frangou, 2004; Euser et al. 2012; Iacono & Malone, 2011; Iacono et al. 2017; Solowij, Michie, & Fox, 1991). Reduced *θ* and *δ* energies and ITPC are also associated with substance use and schizophrenia (Burwell, Malone, Bernat, & Iacono, 2014; Ethridge et al. 2015, 2012; Ford, Roach, Hoffman, & Mathalon, 2008; Harper, Malone, & Iacono, 2019b; Jones et al. 2006; Rangaswamy et al. 2007; Yoon, Malone, Burwell, Bernat, & Iacono, 2013).

### Resting-state EEG power

EEG was recorded while participants were asked to relax with eyes closed for 5 min and listen to soft white noise. We obtained power in *δ*, *θ*, *α*, and *β* frequency bands via a fast Fourier transformation of EEG at the central midline electrode Cz (averaged bilateral ear-lobe signal reference). Alpha power was also calculated from the average of two bipolar parieto-occipital derivations (O1-P7 and O2-P8). Individual differences in resting-state power have been linked to schizophrenia (Hong, Summerfelt, Mitchell, O’Donnell, & Thaker, 2012; Kam, Bolbecker, O’Donnell, Hetrick, & Brenner, 2013; Narayanan et al. 2014; Venables, Bernat, & Sponheim, 2009), alcohol dependence (Kamarajan, Pandey, Chorlian, & Porjesz, 2015; Rangaswamy & Porjesz, 2014), smoking (Rass, Ahn, & O’Donnell, 2016; Su et al. 2017), cannabis use (Ehlers, Phillips, Gizer, Gilder, & Wilhelmsen, 2010; Herning, Better, Tate, & Cadet, 2003; Struve et al. 1999), externalizing (Rudo-Hutt, 2015), and intelligence quotient (Langer et al. 2012; Posthuma, Neale, Boomsma, & de Geus, 2001; Thatcher, North, & Biver, 2005).

### Eye tracking

Participants were asked to fixate on a dot in the center of a computer screen. At variable intervals, a second dot was flashed to either side of the screen and participants were instructed to look in the opposite direction. The *antisaccade* measure is the proportion of trials in which the individual looked toward the light rather than away from it (failure to inhibit their prepotent response). Several studies have suggested antisaccade error rate as an endophenotype for schizophrenia (Calkins, Curtis, Iacono, & Grove, 2004; Calkins, Iacono, & Ones, 2008; Levy, Mendell, & Holzman, 2004; McDowell et al. 2002; Radant et al. 2010).

### Creation of PGS

Summary statistics for drinks per week and regular smoking initiation (binary phenotype of ever being a regular smoker in one’s lifetime, coded as 0 = no and 1 = yes) were obtained from the GWAS and Sequencing Consortium of Alcohol and Nicotine (GSCAN) use; see (Liu et al. 2019) for details.^1^ Summary statistics from the largest GWAS meta-analysis of lifetime cannabis use (binary phenotype of having ever used cannabis, coded as 0 = no and 1 = yes) were obtained (Pasman et al. 2018). Summary statistics for schizophrenia were obtained from a recent GWAS meta-analysis of schizophrenia, the largest of its kind to date (https://walters.psycm.cf.ac.uk/) (Pardiñas et al. 2018). Summary statistics for educational attainment were obtained from the largest to date GWAS meta-analysis of educational attainment (Lee et al. 2018). MCTFR was one of the discovery cohorts in the GSCAN, cannabis, and educational attainment GWAS meta-analyses and, as such, was not included in the set of summary statistics used to create the PGS here.

The final PGS for drinks per week (discovery *N* = 937 381) contained 1 093 636 variants, regular smoking initiation (discovery *N* = 1 225 910; 52.0% of which were cases on average across all meta-analyzed studies) contained 1 093 640 variants, lifetime ever use of cannabis (discovery *N* = 184 765; 28.8% cases) contained 805 738 variants, schizophrenia (discovery *N* = 105 318; 38.6% cases) contained 1 073 315 variants, and educational attainment (discovery *N* = 762 526) contained 1 093 298 variants.

PGSs were calculated using LDPred (Vilhjálmsson et al. 2015), a Bayesian method of PGS calculation that estimates posterior mean causal effect sizes from GWAS summary statistics conditioning on a point-normal mixture distribution for the genetic architecture of effects and a reference sample for LD patterns. MCTFR genotypes were pruned to only those with imputation quality score R^2^*>* 0.7, then further limited to variants with minor allele frequency *>* 0.01 and present in HapMap3 as these reflect the vast majority of common genetic variance and are extensively vetted variants with stable and well-known properties. LDPred was used to calculate beta weights for variants of all significance levels (*p ≤* 1). Individual PGS were then calculated in PLINK 1.9 (Chang et al. 2015).

### Statistical analysis

All statistical analyses were conducted in R (R Core Team, 2019).

First, we calculated twin/family correlations and twin-based heritability (Boker et al. 2011) for each endophenotype. Next, we examined the correlations between PGSs to both evaluate the covariation among the PGS for each phenotype and ensure that the calculated PGSs behave as expected (e.g. substance use PGS relate to each other).

To calculate multivariate endophenotypes, PCA [*psych* R package (Revelle, 2020)] was used to exploit the covariation among endophenotypes and extract a smaller number of meaningful dimensions/components for statistical analysis. Analyzing PCs that account for most of the variance of the observed endophenotypes also reduces the burden of multiple testing relative to testing each of the 11 endophenotypes individually. As a preliminary step prior to PCA, missing endophenotype data was imputed with the regularized iterative PCA algorithm [*missMDA* R package (Josse & Husson, 2016)] using generalized cross-validation to empirically choose the most appropriate imputation method (Josse & Husson, 2012). The algorithm indicated mean imputation as the most appropriate, which has been shown to be a viable option for moderately correlated variables [*r ~* 0.30; (Dray & Josse, 2015)], such as those in the current study (see online Supplementary Fig. S1). We note that the pattern of results was identical when using the pairwise correlation approach (using all complete pairs of observations) to deal with missing data (results not shown). Parallel analysis using both resampled and simulated data (2000 iterations) were used to determine the number of components. Components were retained if the actual data eigenvalue was greater than the corresponding simulated/resampled data eigenvalue. The component structure was obliquely rotated (Promax) to facilitate interpretation and scores were calculated for statistical analyses.

The main analyses of interest, that is, testing the association between each PGS (independent variable) and each multivariate endophenotype PC (dependent variable) were calculated using a rapid feasible generalized least-squares (RFGLS) regression method [RFGLS R package (Li, Basu, Miller, Iacono, & McGue, 2011)] to account for dependency among parents, monozygotic (MZ) twins and dizygotic (DZ) twins and calculate appropriate standard errors in the presence of clustered data. To evaluate uncertainty around effect sizes and determine the significance of the standardized beta (*β*) estimates, we used the *car* R package (Fox & Weisberg, 2019) to conduct nonparametric residual bootstrapping (5000 random draws) of the regression models and the *boot* package (Canty & Ripley, 2021) to compute bias-corrected and accelerated 95% confidence intervals (CIs) [for a technical discussion, see (van der Leeden, Meijer, & Busing, 2007)].

## Results

### Endophenotype descriptions

Table 1 contains descriptive statistics for each endophenotype. In all cases, the within-family mother–father correlations were negligible and offspring–parent correlations were small. Endophenotypes were moderately to strongly heritable, as evidenced by MZ correlations being at least approximately twice the DZ correlations and the twin-based heritability point estimates.

### Associations among PGSs

A pattern of shared genetic liability was observed across the PGSs, as shown in Table 2. As expected, the three substance use PGSs were positively correlated, and all were positively correlated with the schizophrenia PGS. The education attainment PGS was negatively correlated with the regular smoking PGS but positively correlated with the cannabis use PGS, a pattern consistent with other studies (Pasman et al. 2018; Wedow et al. 2018).

Notably, the correlation between drinks per week and regular smoking PGS was in the same direction as the genetic correlations between these phenotypes [as reported previously; (Liu et al. 2019)], albeit lower in magnitude. This is expected since the present correlations reflect the covariance between PGSs, not the latent additive genetic covariance between two phenotypes, and supports the construct validity of the PGSs.

### PCA-based multivariate endophenotypes

**Fig. 1.**
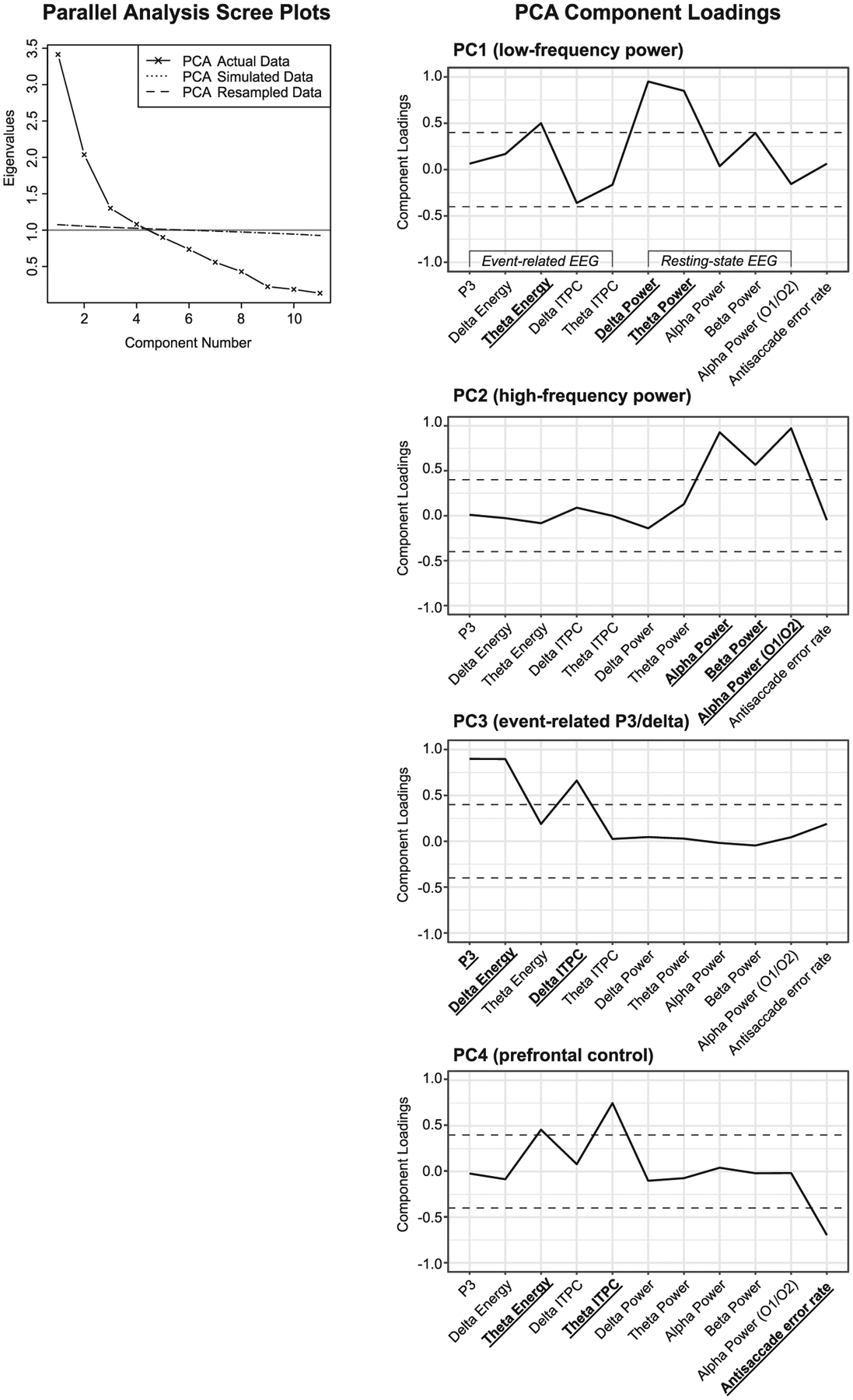
Left. Scree plots of the principal component analysis (PCA) eigenvalues estimated from the actual (observed) data and eigenvalues from two forms of parallel analysis (simulated and resampled data). The plot provides empirical support for retaining four PCs as the actual data eigenvalues were greater than the simulated/resampled eigenvalue for components 1–4 but not 5. The gray line along the *y*-axis demarcates the traditional Kaiser’s eigenvalues greater than one rule, which also supports four components. Right. Profile plots of the component loadings (Promax oblique rotation) for each endophenotype on PCs 1–4. Loadings >|0.40| (illustrated by the dashed line) were used in the interpretation of the components; endophenotypes with loadings ≥|0.40| are indicated in bold on the *x*-axis. ITPC, intertrial phase consistency.

The parallel analysis supported extracting four components explaining a total of 72% of the variance across endophenotypes (Fig. 1) with each respective component explaining 21, 20, 19, and 11% of the variance. Component loadings are shown in Fig. 1; loadings ≥|0.40| were used to interpret the PCs (i.e. multivariate endophenotypes). PC1 primarily indexed *low-frequency power* (strongest loadings: resting-state *δ*/*θ* power), PC2 captured *high-frequency power* (strongest loadings: resting-state *α*/*β* power), PC3 indexed *event-related P3/δ* (strongest loadings: P3, *δ* energy, and *δ* ITPC), and PC4 primarily captured endophenotypes related to *prefrontal control* (strongest loadings: antisaccade error rate, *θ* ITPC, and *θ* energy). *θ* energy cross-loaded on PC1 and PC4. All four multivariate endophenotypes were heritable (Table 3).

### Associations between PGSs and multivariate endophenotypes

The association between multivariate endophenotypes and PGSs are presented in Table 4. Four associations were statistically significant. Polygenic risk for schizophrenia was negatively predictive of PC3 scores (i.e. the event-related P3/*δ* component). Three significant associations were observed for the prefrontal control PC4 (*θ*/antisaccade): the drinks per week and regular smoking PGSs were negatively associated with PC4 scores, whereas the educational attainment PGS was positively associated with PC4 scores^2^ No significant associations were observed for the low- or high-frequency resting-state power multivariate endophenotypes or the cannabis use PGS.

## Discussion

In this current report, we substantially extended our previous work by testing the relationship between multivariate endophenotypes and up-to-date PGSs spanning multiple domains. Our previous investigation, designed to test the individual predictive utility of 8 of the current 11 endophenotypes with a schizophrenia PGS constructed using weights from a relatively large schizophrenia GWAS meta-analysis (Schizophrenia Working Group of the Psychiatric Genomics Consortium, 2014) was unsuccessful (Liu et al. 2017). In the current report, we adopted several approaches aimed at increasing the power to identify significant PGS–endophenotype associations. We utilized up-to-date PGSs for alcohol use, regular smoking, cannabis use, schizophrenia, and educational attainment calculated with well-powered GWAS meta-analysis summary statistics (Lee et al. 2018, 2019; Pardiñas et al. 2018; Pasman et al. 2018) and more sensitive statistical methods (LDPred), which should in theory produce more robust PGSs with greater reliability and explanatory power relative to our prior report. We also examined additional candidate endophenotypes not tested in our previous report (i.e. *δ*/*θ* energy and ITPC). Finally, rather than testing each endophenotype in isolation, a PCA-based multivariate endophenotype approach was used to leverage the combined explanatory influence across 11 candidate endophenotypes. We observed significant associations between PGSs and multivariate endophenotypes reflecting event-related EEG activity and prefrontal control-related endophenotypes. Specifically, an event-related P3/*δ* component was negatively associated with schizophrenia PGS, whereas a prefrontal control component was negatively related to drinks per week and regular smoking PGSs but positively associated with educational attainment PGS. In contrast, no significant effects were found for multivariate endophenotypes of low- or high-frequency resting-state power or the cannabis use PGS. Findings offer novel preliminary evidence linking psychophysiological multivariate endophenotypes to polygenic liability in psychiatric/psychological phenotypes.

Within a construct validation framework, aside from simply sharing genetic variance with a phenotype, endophenotypes are expected to show robust associations with specific genetic variants (Gottesman & Gould, 2003; Iacono et al. 2017). The use of endophenotypes to identify single genes/variants related to psychological phenotypes has not yielded much success (Iacono et al. 2017). In contrast to examining the influence of a single allele, it has been suggested that polygenic approaches may increase the chance of identifying endophenotypes, and therefore potential biobehavioral mechanisms, related to genetic liability for a psychiatric disorder or psychological phenotype (Bogdan et al. 2018). In a similar vein, by jointly leveraging the shared and unique genetic variance across several measures, multivariate endophenotypes may better index the multiple biological and cognitive risk pathways influencing a phenotype than any single endophenotype alone (Frederick & Iacono, 2006; Gottesman & Gould, 2003). Informed by these suggestions, we observed several novel findings of statistically significant associations between PGS and multivariate endophenotypes. An event-related parietal P3/*δ* component was negatively related to schizophrenia polygenic risk. A prefrontal control component indexing event-related frontal *θ* and antisaccade performance had negative associations with PGSs for both drinks per week and regular smoking initiation, and a positive association with educational attainment PGS. While effect sizes were small in magnitude, we believe that these findings can serve as potentially promising leads for future research using further refined PGS and multivariate endophenotypes in even larger samples, such as the EEG workgroup of the ENIGMA (Enhancing NeuroImaging Genetics through Meta Analysis) consortium (Thompson et al. 2017), and we discuss their potential implications below.

Polygenic risk for schizophrenia was negatively correlated with a multivariate endophenotype primarily reflective of parietal P3 amplitude and *δ* energy and ITPC to target stimuli during a visual oddball task. This pattern is consistent with previous literature demonstrating a genetic association between reduced P3 and schizophrenia (Ford, 1999; Jeon & Polich, 2003) and reduced *δ* energy/ITPC in individuals with schizophrenia (Ford et al. 2008). In neurocognitive terms, P3 and *δ* activity elicited by rare target detection are correlates of decision-making and signal-matching processes, such as evaluating whether a stimulus classification and the associated chosen response choice is appropriate (Başar-Eroglu, Başar, Demiralp, & Schürmann, 1992; Cooper, Darriba, Karayanidis, & Barcelo, 2016; Harper, Malone, & Iacono, 2017; Verleger & Śmigasiewicz, 2016). Anomalies in these EEG correlates may be part of a constellation of traits associated with polygenic risk for schizophrenia, and if confirmed by future work, offer further support of these measures as endophenotypes for schizophrenia. It should be noted that these effects are in contrast to our previous report that found no significant associations with P3 (Liu et al. 2017). This is likely attributable to three key differences in the current report: (1) relative to the PGS used in our previous report, we used an updated PGS from the largest schizophrenia GWAS to date (Pardiñas et al. 2018) that identified more significant associated loci (145 compared to 108) and explained more variance in schizophrenia liability (5.7% compared to 3.4%); (2) the use of a more powerful PGS calculation analytic tool (LDPred), likely producing a more robust/predictive PGS; and (3) the multivariate endophenotype approach combining several schizophrenia-related endophenotypes, including novel measures of *δ* energy and ITPC alongside P3.

A multivariate endophenotype capturing prefrontal control-related measures (*θ* energy and ITPC, antisaccade error rate) was associated with PGS for alcohol consumption, regular smoking, and educational attainment. Frontal *θ* activity is thought to reflect a reactive mechanism related to successful attentional allocation, orienting, and control-related prefrontal cortex processes (Başar-Eroglu et al. 1992; Cavanagh & Frank, 2014; Clayton, Yeung, & Kadosh, 2015; Harper et al. 2017), and is a candidate endophenotype for (poly)substance use (Harper et al. 2019b; Rangaswamy et al. 2007). Antisaccade performance has been linked to prefrontal inhibitory control (Hutton & Ettinger, 2006), and some evidence suggests it indexes risk for substance use/behavioral disinhibition (Iacono, 1998; Young et al. 2009). The current findings, if confirmed by future research, suggest that individual differences in a multivariate endophenotype related to frontal executive functioning may index the polygenic risk for both regular smoking and the number of alcoholic drinks per week, which may have significant public health implications given the prevalence of alcohol and nicotine use (Substance Abuse and Mental Health Services Administration, 2020). The lack of significant cannabis use PGS effect may reflect a level of differentiation between substances and endophenotypes despite moderate genetic overlap among substances in this sample (Table 2) and others (Jang et al. 2020; Liu et al. 2019; Pasman et al. 2018). The cannabis GWAS identified fewer significant variants compared to the similarly sized schizophrenia or much larger alcohol/smoking GWASs; cannabis-related variants might have very small effect sizes (like those for alcohol/smoking) and a significantly larger discovery sample may be needed to improve precision/power of the cannabis PGS to detect endophenotype effects. The prefrontal control multivariate endophenotype was also positively correlated with educational attainment PGS, potentially reflecting an improved ability to deploy frontal attentional and executive control processes in those with a higher polygenic load for completing more years of education.

While not a primary focus of this report, we observed several novel significant cross-trait associations between PGSs. For example, positive correlations were found between schizophrenia, alcohol, regular smoking, and cannabis use PGSs, which suggests a common genetic basis (shared loci) indicative of a general vulnerability toward all four phenotypes (Jang et al. 2020). Increased polygenic risk for regular smoking was related to decreased polygenic load for educational attainment, which is interesting given that both were associated (in opposing directions) with the prefrontal control multivariate endophenotype.

We acknowledge that the potential utility of the current findings is limited given the small effect sizes between PGSs and endophenotypes. However, this was unsurprising as the cross-trait explanatory variance of PGSs is often small even for the phenotypes from which they are derived. For example, as reported in the original articles, the phenotypic variance explained by PGSs was 1 and 4% for drinks per week and regular smoking, respectively (Liu et al. 2019) and highest at ~12% for educational attainment (Lee et al. 2018). The relationship between the PGS and a brain measure is likely expected to be even smaller, as distant cross-domain correlations are expected to be lower than closely related within-domain measures (Campbell & Fiske, 1960), consistent with what was found here. The significant PGS-multivariate endophenotype associations observed in this report all had absolute standardized *β* estimates of ~0.03, explaining 0.10–0.12% of the variance (Buse, 1973), whereas PGS–PGS associations explained up to 5.66%. As suggested in guidelines proposed in a recent review on effect sizes in psychological science (Funder & Ozer, 2019), small effect sizes found in large samples are to be expected, can have large downstream causal effects, and are likely more believable than the inverse. It may be that other psychophysiological measures or further refined PGS with even larger discovery samples and stronger analytic methods may explain more production of larger effect sizes, but this remains to be seen. Nevertheless, these findings show that progress is being made in linking endophenotypes to specific polygenic influences. We note that endophenotypes have additional potential utility beyond identifying specific PGS links, such as prospectively predicting phenotypic expression (e.g. substance use initiation, see Anokhin and Golosheykin, 2016; Harper et al. 2019a) or informing brain mechanisms that may potentially identify system-level targets for treatment responses or environmental interactions (Bogdan et al. 2018; Iacono et al. 2017) in a similar fashion as to how GWAS has helped understand how certain tissues or genes may be associated with a trait.

Another potential limitation may be PGS imprecision. By aggregating across many variants not associated with the endophenotype but rather only with the phenotype, PGSs may contain ‘noise’ that may downwardly bias its association with an endophenotype. Another issue relevant to the current state of GWAS research is the lack of a large sample non-European GWAS meta-analyses. The GWAS meta-analyses statistics used here are all based on individuals of European descent, thus limiting the target population to only Europeans. More research is needed in non-European populations to generalize the effectiveness of both the PGS, which may vary in part by ancestral allele frequency differences, and endophenotypes to better understanding the biological underpinnings of these complex traits.

The current results represent meaningful progress in linking polygenic liability for schizophrenia, alcohol use, regular smoking, and educational attainment to multivariate psychophysiological endophenotypes of decision-making (P3/*δ*) and prefrontal control (*θ*/antisaccade). While at present endophenotypes may not explain large amounts of polygenic variance in psychopathology or psychological traits, these results are an encouraging step forward. Future studies will likely benefit from leveraging a large collection of relevant endophenotypes, each likely accounting for a small amount of variance, in a multivariate fashion to better understand the polygenic risk associated with a single trait.

## Supplementary Material

Suppl 1

## Figures and Tables

**Table 1.** Summary statistics for the endophenotypes

Endophenotype	*n*	Mean age (years)	% Female	Within-family correlations					Twin-based heritability (95% CIs)
				Mother-father	Off.-mother	Off.-father	MZ twins	DZ twins	
Event-related EEG									
P3	4155	29.01	44	0.00	0.25	0.19	0.64	0.39	0.48 (0.30–0.66)
*δ* energy	4138	29.02	43	−0.03	0.18	0.16	0.61	0.34	0.52 (0.33–0.65)
*θ* energy	3420	29.27	50	−0.03	0.17	0.20	0.65	0.17	0.63 (0.56–0.68)
*δ* ITPC	4153	29.01	43	−0.04	0.11	0.04	0.46	0.20	0.46 (0.31–0.51)
*θ* ITPC	3427	29.28	50	−0.05	0.09	0.06	0.41	0.16	0.40 (0.24–0.47)
Resting-state EEG									
*δ* power	3938	28.75	44	−0.12	0.21	0.08	0.56	0.24	0.56 (0.42–0.61)
*θ* power	3938	28.75	44	−0.07	0.22	0.15	0.73	0.36	0.73 (0.57–0.76)
*α* power	3938	28.75	44	0.07	0.28	0.31	0.85	0.45	0.63 (0.78–0.86)
*β* power	3938	28.75	44	0.00	0.36	0.23	0.85	0.38	0.85 (0.75–0.87)
*α* power (O1/O2)	3956	28.77	44	0.05	0.30	0.28	0.80	0.42	0.77 (0.61–0.82)
Eye tracking									
Antisaccade	4457	28.98	44	0.04	0.24	0.17	0.53	0.18	0.52 (0.43–0.56)

Off., offspring; MZ, monozygotic; DZ, dizygotic.

**Table 2.** Correlations among PGSs

	1	2	3	4	5
1. Drinks per week	–	–	–	–	–
2. Regular smoking	**0.236 (0.209–0.263)**	–	–	–	–
3. Cannabis use	**0.133 (0.106–0.161)**	**0.220 (0.192–0.247)**	–	–	–
4. Schizophrenia	**0.042 (0.014– 0.070)**	**0.142 (0.114–0.171)**	**0.116 (0.089–0.144)**	–	–
5. Educational attainment	**0.036 (0.007–0.064)**	−**0.238 (**−**0.265 to** −**0.210)**	**0.182 (0.155–0.211)**	−0.019 (−0.047 to 0.010)	–

*Notes:* Nonparametric bootstrap 95% confidence intervals are presented under correlation point estimates. Intervals that did not overlap with zero were considered significant and are bolded.

**Table 3.** Within-family correlations and twin heritability estimates for the multivariate endophenotypes

Multivariate endophenotype PC scores	Within-family correlations					Twin-based heritability (95% CIs)
	Mother-Father	Off.-mother	Off.-father	MZ twins	DZ twins	
PC1 (low-frequency power)	−0.07	0.17	0.10	0.65	0.28	0.65 (0.56–0.68)
PC2 (high-frequency power)	0.06	0.26	0.23	0.76	0.41	0.68 (0.54–0.78)
PC3 (event-related P3/*δ*)	0.00	0.20	0.17	0.58	0.30	0.54 (0.38–0.62)
PC4 (prefrontal control)	0.02	0.18	0.13	0.50	0.23	0.50 (0.37–0.54)

PC, principal component; Off., offspring; MZ, monozygotic; DZ, dizygotic.

**Table 4.** Associations between multivariate endophenotypes and PGSs

Multivariate endophenotype PC scores	PGS				
	Drinks per week *β* (95% CI)	Regular smoking *β* (95% CI)	Cannabis use *β* (95% CI)	Schizophrenia *β* (95% CI)	Educational attainment *β* (95% CI)
PC1 (low-frequency power)	0.001 (−0.027 to 0.029)	0.006 (−0.022 to 0.035)	−0.005 (−0.032 to 0.022)	−0.008 (−0.036 to 0.019)	0.013 (−0.015 to 0.041)
PC2 (high-frequency power)	0.016 (−0.012 to 0.044)	0.002 (−0.026 to 0.030)	0.000 (−0.029 to 0.028)	−0.011 (−0.040 to 0.017)	0.014 (−0.015 to 0.041)
PC3 (event-related P3/*δ*)	−0.011 (−0.040 to 0.016)	−0.006 (−0.033 to 0.022)	−0.010 (−0.039 to 0.019)	−**0.032 (**−**0.059 to** −**0.003)**	0.022 (−0.006 to 0.049)
PC4 (prefrontal control)	−**0.034 (**−**0.063 to −0.006)**	−**0.032 (**−**0.061 to** −**0.005)**	−0.008 (−0.036 to 0.019)	−0.017 (−0.044 to 0.012)	**0.031 (0.003–0.058)**

PC, principal component.

*Notes:* Standardized beta estimates (*β*) with nonparametric bootstrap 95% confidence intervals (CIs) that did not overlap with zero were considered significant and are bolded. Using the formula for generalized least squares proposed by Buse (1973), the *R*^2^ for significant effects ranged from 0.10% for PC3 and regular smoking/educational attainment to 0.12% for PC3-schizophrenia and PC4-drinks per week.

## Footnotes

1 Although several nicotine use polygenic scores were present in GSCAN (Liu et al., 2019), the decision was made to investigate only the regular smoking initiation PGS in the current report for several reasons. As indicated in the discovery GWAS paper from which these measures were derived (Liu et al., 2019), the three other smoking PGS (cigarettes per day, smoking cessation, and age of smoking initiation) were all highly pleiotropic with the regular smoking initiation PGS. Furthermore, regular smoking initiation had a larger number of associated total loci (278) and non-pleiotropic loci (138) than the other smoking PGS (total loci range: 40–72; non-pleiotropic loci range: 0–8). Given this, and the desire to reduce the burden of multiple testing, the regular smoking initiation PGS was chosen for analysis.

2 While the primary analyses focused on the multivariate endophenotypes, *post hoc* follow-up analyses further explored the significant PGS-PC findings by examining associations between PGSs and the constituent endophenotypes loading most strongly on the PCs (i.e. P3 and δ power/ITPC for PC3, θ power/ITPC and antisaccade for PC4). Results are presented in the Supplement. The pattern of effects was largely similar between the individual and multivariate endophenotypes, albeit more consistent for the multivariate endophenotypes.
