# Supplementary material for: Using multivariate endophenotypes to identify psychophysiological mechanisms associated with polygenic scores for substance use, schizophrenia, and education attainment
Source: Psychol Med. 2021 Mar 18;52(16):3913–23. doi: 10.1017/S0033291721000763 (PMC8448784; doi:10.1017/S0033291721000763)

Using multivariate endophenotypes to identify psychophysiological mechanisms associated with polygenic scores for substance use, schizophrenia, and education attainment

Supplementary Information

Correlations among endophenotypes

As shown in Figure S1, correlations among individual endophenotypes were generally modest to moderate in size, and correlations between endophenotypes within the same task/domain (e.g., resting-state power) were stronger than those from different tasks/domains (e.g., antisaccade and P3). These correlations supported our use of the PCA-based multivariate endophenotype approach to capture the covariation among endophenotypes and reduce dimensionality.

Post-hoc follow-up polygenic score (PGS) – endophenotype analyses

The significant PGS-multivariate endophenotype effects reported in Table 4 were decomposed to examine associations between PGSs and those individual endophenotypes loading most strongly on the PC3 (P3/delta) and PC4 (prefrontal control) multivariate endophenotypes. Specifically, the individual endophenotypes evaluated were event-related P3, delta energy, and delta intertrial phase consistency (ITPC) for the main PC3-schizophrenia PGS finding, and event-related theta energy, theta ITPC, and antisaccade error rate for the main PC4 associations with drinks per week, regular smoking initiation, and educational attainment PGSs.

Results are shown in Figure S2. The pattern of effects was largely similar between the individual and multivariate endophenotypes. The schizophrenia PGS had a significant negative association with all three PC3 constituents (i.e., event-related P3 and delta energy/ITPC), consistent with the notion that oddball P3 and delta oscillatory activity index similar underlying processes. Results for the individual event-related theta and antisaccade endophenotypes were all

in the expected directions, with greater alcohol/nicotine PGS associated with lower theta and greater antisaccade error rate; the inverse of that pattern was observed for the educational attainment PGS. However, significance was more mixed for the individual theta and antisaccade error rate endophenotypes relative to the multivariate prefrontal control PC4 findings, underscoring the utility and increased power afforded by the multivariate endophenotype approach.

Supplemental Figure Captions

Figure S1. Zero-order correlations among the endophenotypes and the principal component analysis (PCA) multivariate endophenotype component scores (Promax oblique rotation). All correlations were significant at a false discovery rate of $q < 0.05$ except those cells colored in white. Correlation coefficients that are underlined indicate the endophenotypes that loaded most strongly on the respective PC (e.g., P3, delta energy, and delta intertrial phase consistency [ITPC] for PC3) in the PCA.

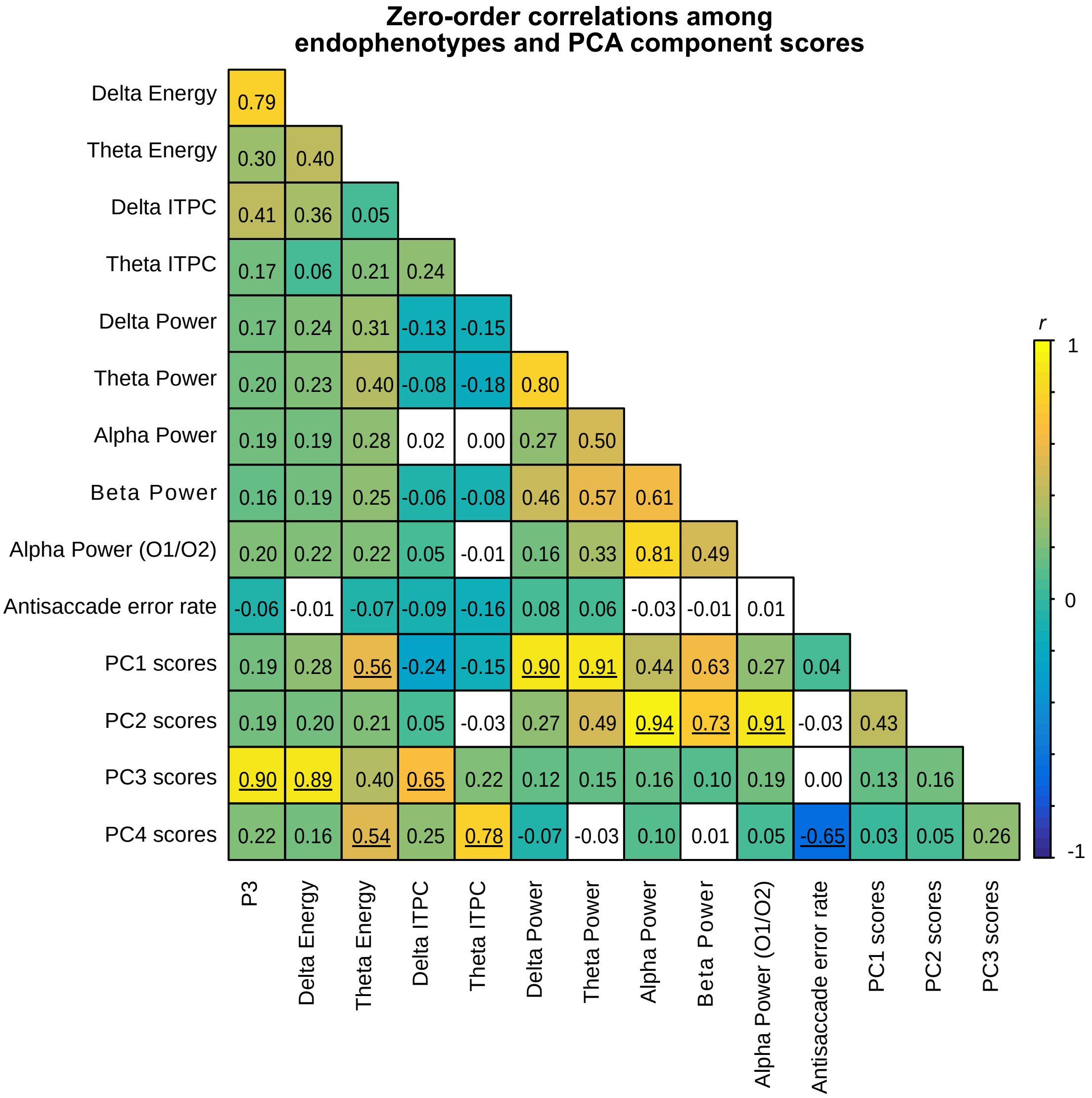

Figure S2. Post-hoc follow-up analyses testing the association between polygenic scores (PGSs) and individual endophenotypes. Plots show the standardized beta (β) parameter estimates (points) and nonparametric bootstrap 95% confidence intervals (CIs; whiskers) for the association between each PGS and the multivariate endophenotypes PC3 and PC4 (i.e., the same results presented in Table 4) and their main constituent endophenotypes. Separate models were computed for each PGS-endophenotype pair.

Abbreviations: ITPC, intertrial phase consistency.

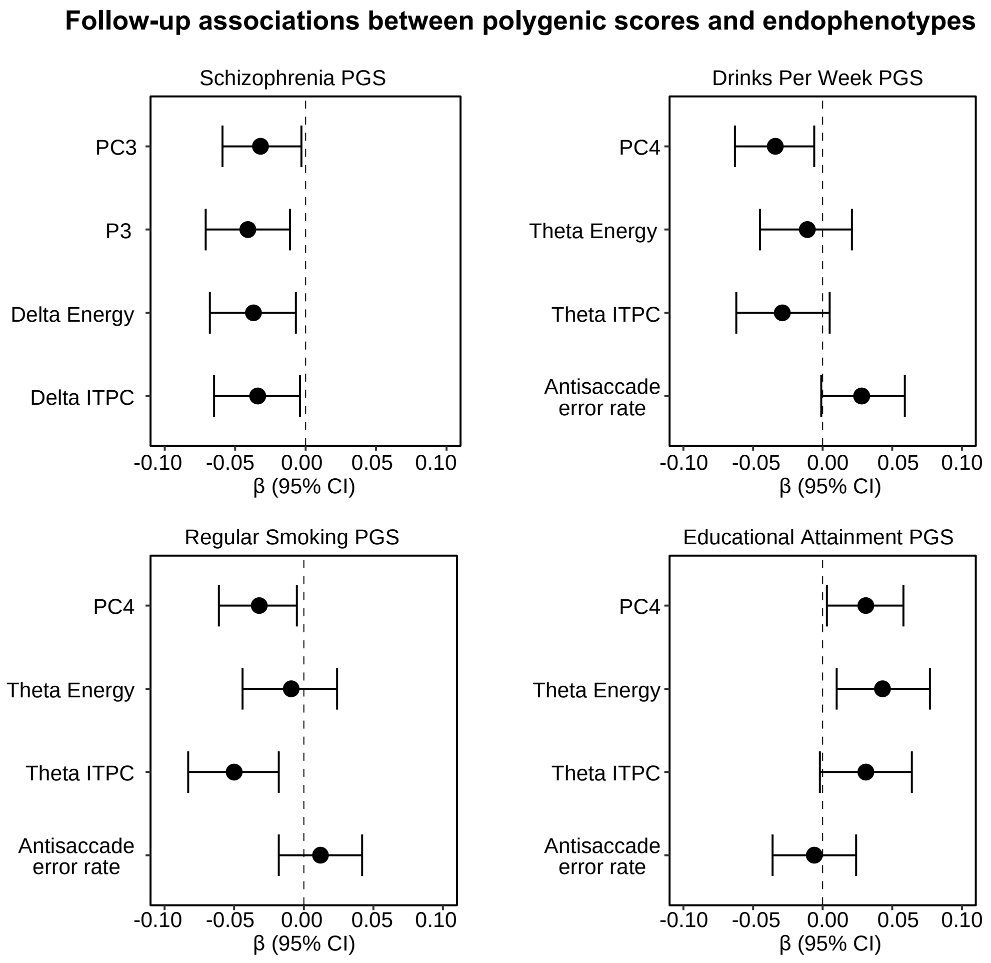

Supplement: Supplementary file 1 [file S0033291721000763sup001.pdf]
